# ‘Is it in your basic personality?’ Negotiations about traits and context in diagnostic interviews for personality disorders

**DOI:** 10.1177/13634593221094701

**Published:** 2022-05-24

**Authors:** Maarit Lehtinen, Liisa Voutilainen, Anssi Peräkylä

**Affiliations:** University of Helsinki, Finland; University of Helsinki, Finland; University of Helsinki, Finland; Freiburg Institute for Advanced Studies, Germany

**Keywords:** clinician-patient interaction, conversation analysis, discursive psychology, personality disorders, psychiatric interview

## Abstract

What does it mean to claim that somebody’s personality is disordered? The aim in this paper is to examine how the process of diagnosing personality disorders (PD) unfolds on a practical level. We take an in-depth look at PD interviews, paying close attention to the occasional discrepancies in the clinicians’ and the patients’ approaches to generalising the behaviour of patients to describe their personality. Clinicians are guided by the medical model and structured interviews in their approach. We regard the interview situation as interplay between the institution, the clinician and the patient – and the final diagnosis as an interactional construction between them. Our data consists of video-recorded interviews in Finland with 10 adult patients and three psychiatric nurses. The collection was compiled from 22 excerpts in which the participants orient differently to the generalisability of personality traits. Our observations show that, in these interviews, patients frequently make sense of their behaviour differently from what is expected – not as a reflection of their personality traits, but as an outcome of many situational factors. Our understanding leads us to emphasise the importance of making visible the practices that shape the diagnostic process in psychiatry.

## Introduction

As Foucault (2006) already pointed out in the ‘70s, psychiatric evaluation seeks stable and regular patterns in a patient’s behaviour. Patients often see their behaviour in a different way, explaining it in terms of life circumstances and social relations. Drawing up from the interactive theory of Habermas (1971), Mishler (1984) uses the term ‘voice of lifeworld’ to describe the patients’ orientation. The counterpart is ‘voice of medicine’, meaning the biomedical model as the guiding orientation in the interaction.

Using Mishler’s theory as a conceptual framework, we investigate real-life psychiatric encounters. Although the importance of the patient’s subjective experience is commonly acknowledged, there has been little research on how clinicians adapt this to their psychiatric evaluation (Savander et al., 2019). Our focus in this paper is on the discrepancies in how clinicians and patients describe generalised patterns in patient’s behaviour and personality in the context of diagnosing personality disorders. Our analysis shows how clinicians turn patients’ descriptions of context-specific experiences into the psychiatric language of personality traits.

This study offers a sociological perspective on psychiatric practice, leaning methodologically on conversation analysis (CA) (Antaki, 2011) and discursive psychology (Edwards and Potter, 1992). CA facilitates the sensitive description of participant’s orientations towards the social situation they collaboratively create (Schegloff, 1991). There is a strong tradition of CA research in psychotherapeutic practice (for a review see Peräkylä, 2019). Recently, it has also been applied in the study of psychiatric encounters.

In a CA based study on psychiatric encounters, Weiste et al. (2018) shed light on the voice of medicine and the voice of lifeworld in patients’ diagnostic talk. Although the patients tended to explain their symptoms in terms of life experiences, they also used diagnostic categories. They referred to the diagnoses either when explaining the life experiences that caused them or when reducing their own responsibility for their behaviour (Weiste et al., 2018).

Interactional research is an emergent field in psychiatry. Most studies focus on decision-making on medication (e.g. Bolden et al., 2019; Quirk et al., 2012; Thompson and McCabe, 2018). To our knowledge, no research thus far has focussed on the interaction during structured PD interviews (see, however, Peräkylä et al. in review on unstructured interviews). Our aim in this paper is to investigate both how the patients account for their behaviour and the reasoning clinicians use in their talk for diagnostic purposes.

### Psychiatric interviews

A psychiatric interview is a variant of a medical interview, and follows a similar question-answer format. However, there are differences, in that there is no clear bodily syndrome to discover. Ziółkowska (2009) describes a psychiatric interview as a social situation in which the participants create meanings for events and thus construct reality. Hence, the interview is not a unidirectional act in which the patient informs the clinician; they negotiate, fix and change the interpretations together during the interaction (Ribeiro and de Souza Pinto, 2006).

Interviewers control the topic, directing the talk by means of questions, silences and response tokens. They decide which part of the answer leads to further processing - thus they cannot remain neutral in a strict and formal sense (Rapley, 2001). The questions define the information that is relevant in the answers, thereby influencing the final diagnosis (Ziółkowska, 2009). A structured interview − such as the Structured Clinical Interview for DSM-IV Axis II Personality Disorders, SCID-II (First et al., 1997) − powerfully directs the conversation and ties the interviewer to the set of questions. However, interviewers have their own unique influence on the process in terms how they receive the patients’ answers and ask follow-up questions.

Clinicians face the challenge of balancing between two different approaches in their work (Ziółkowska, 2009). On the one hand they should listen to the narratives of patients talking about their experiences, and on the other hand they have to work within the medical framework in which the interview is a technique used to obtain the factual information required for diagnosis. Therefore, in Mishler’s terminology, they need to translate the voice of lifeworld into the voice of medicine. Ziółkowska (2012) also found that, in the medical framework, psychiatrists often transform patients’ context-bound behaviour into an inner and stable trait.

McGann (2011) claims that symptom checklists detach symptoms from their contexts and make it more difficult to evaluate patients’ meaningful reactions. According to Georgaca (2013), one way in which professionals turn clinical interviews into psychiatric reports is by selecting only the information that supports the psychiatric formulation. To fit the patient’s experiences into the standardised model of reporting, for example, they transform everyday terms into psychiatric language and blur the professional’s influence on the process.

Nurses carry out most of the diagnostic work in our data, whereas in most earlier research, doctors have been responsible for the diagnostic screening. Hence, the setting is not completely comparable in that there might be some variation among the professions. The question of whether psychiatric nurses carry out the interviewing differently from psychiatrists remains open for the upcoming empirical research.

### The evaluation of personality disorders

We focus on how a clinician and a patient go through the SCID-II interview. Personality disorders (PDs) are understood to be ingrained, maladaptive, long-lasting and rigid traits that differ significantly from cultural expectations. These enduring patterns should manifest as inflexible responses to a broad range of personal and social situations. A PD diagnosis requires that the traits cause subjective suffering or problems in functioning (Clark, 2007). Usually, the self-related troubles go hand in hand with troubles in relationships. The relational troubles might also affect interaction with health care professionals (Lawn and McMahon, 2015). It is especially this interpersonal nature of PDs that make them so interesting for sociological scrutiny.

There are two main manuals for psychiatric diagnoses in western countries: the DSM (American Diagnostic and Statistical Manual of Mental Disorders) by American Psychiatric Association (2013) and the ICD (International Statistical Classification of Diseases and Related Health Problems) by World Health Organization (WHO, 2018). The 11th version of the ICD will only be applied from 2022 on, hence the 10th version was still in place during our data collection. It is noteworthy that in the new version the PD diagnoses will be changed from categorical model into a continuum of traits. For now, the categorical model is still valid in both the ICD and the DSM manuals.

Even though SCID-II yields psychiatric diagnoses that are consistent with DSM, it is widespread also in countries using the ICD; the DSM diagnoses are typically converted to their equivalent in the ICD (Boberg et al., 2020). Clinicians also widely use SCID-II in Finland. The interview starts with open questions, which are followed by 140 questions that require a yes/no answer, such as ‘Are you afraid to try new things?’ and ‘Do you flirt a lot?’. The questions form modules of different categories of personality disorder. An interviewer may ask extra questions in order to confirm the diagnosis. A PD diagnosis requires an overall clinical judgement and it should not be based solely on SCID-II.

In defining the disorder, both manuals concentrate on the individual. The description of PDs as ‘deeply ingrained patterns’ in ICD-10 (WHO, 1992) implies some independence from the patient’s current life situation. The instructions do not explain how to separate patterns of conduct from the varying life circumstances. Therefore, interviewers have to use their own discretion in separating behaviours that are meaningful in their context from behaviours that are indicative of PD. SCID-II, like any other interview, is based on interaction between two parties, but the diagnostic criteria concentrate solely on the patient and do not take the doctor-patient interaction into account (Ribeiro and de Souza Pinto, 2006). As far we know, no studies thus far have concentrated on the interactional side of SCID-II. We argue that this qualitative aspect of psychiatric interviews requires more attention.

### The contested concept of personality disorder

The PD diagnosis itself is a highly problematised issue. Manning (2000) points out that even among psychiatric diagnoses, which are generally contested constructs, PDs are an especially challenging category. There are issues with poor construction validity, high comorbidity and unclarity regarding underpinning neurobiological mechanisms. For instance, regarding the borderline PD there is not a clear agreement whether it should be treated as a PD or as a trauma response condition (Duff et al., 2020). In addition, Pickersgill (2012) has shown in his analysis regarding the history of antisocial PD how the terms and definitions are prone to change within time and depending on the personal influence each DSM committee group member has on the decision process.

Despite the common idea of PDs as firm personality styles, on many occasions the conditions do not seem very stable: actually, only less than half of the patients meet the diagnostic criteria every month of the year, and in a longer surveillance many have stopped meeting the criteria altogether (Clark, 2007). It seems that the stability is only moderate, at most.

Clark (2007) points out that the PD criteria themselves vary in relation to stability: some of them refer to more and some of them refer to less stable traits. She also mentions that even though the broader personality dimensions remain rather stable, the affect levels vary more because they associate to positive and negative life events. This means that the quality of personality is more permanent while the quantitative intensity varies more.

## Data

Our data consists of video-recorded SCID-II interviews involving 10 adult patients and three female psychiatric nurses. The recordings were made in a psychiatric outpatient clinic in Southern Finland. The patients had been referred to the evaluation team whose task it is to decide about the coming treatment. Patients meet a psychiatric nurse about from three to five times during the evaluation period. First, the nurse obtains an overall view of the patient’s situation and symptoms. Later, SCID-II interviews are carried out with some patients who might have a PD. Nurses also use it for the purpose of differential diagnostics, such as ADHD.

After the evaluation period, the nurse reports the findings to a psychiatrist who makes the final decision regarding diagnosis and treatment. Depending on the situation, some patients also meet the psychiatrist, but many do not. The information about the personality-disorder screening is part of the overall evaluation and may have implications regarding the treatment plan.

It is common in the clinic that patients first fill in a self-assessment questionnaire for SCID-II. The nurses then interview them based on their responses, concentrating mainly on the questions to which the answer is in the affirmative. The nurses ask the questions again to make sure that the patients have understood them correctly. There is a personality questionnaire SCID-5-pd associated with the newest version of the DSM. However, in our data the older version SCID-II is still in use (the newest manual was translated into Finnish only recently), but there is no remarkable difference between the two questionnaire forms.

We restricted our analysis to the SCID-II part of the evaluation. We received informed consent from all the patients and clinicians involved. Approval for the study was obtained from the ethical committee of the Hospital District of Helsinki and Uusimaa. The videos were used to achieve maximal information of the interaction process. There were two tiny GoPro cameras set up in the room, one directed towards the patient and the other one towards the psychiatric nurse.

We analysed the data based on the original Finnish version. The first author of the article transcribed the extracts of the collection in the University of Helsinki. The data was transcribed by using notations that were originally created by Jefferson (2004). All the writers of this article were involved in the analysis process. Afterwards, the extracts were translated into English. Small changes were made to the original extracts to ensure the anonymity of the patients. The data was handled following the ethical and information security protocols, such as GDPR regulations.

### Data availability statement

Due to ethical reasons the data cannot be made freely available. The anonymised data that support the findings of this study are available from the corresponding author upon reasonable request.

## Methods

The focus in discursive psychology is on the social organisation of speech: how people construct accounts and express psychological phenomena depending on the interactional contexts (Edwards and Potter, 1992). The basic assumption is that people are always involved in social interaction when they are talking, which affects how they express themselves, and hence it is not possible to know what they ‘really think’ (Goodman, 2017). The aim in discursive psychology is thus to investigate how and when participants bring up and construct certain identities in conversation. We adopt this perspective in the present paper in analysing how the participants used a description of mental states for purposes of self-presentation and self-evaluation (Potter and Wiggins, 2017).

CA focuses on detailed microanalysis of interaction. Antaki (2011) defines CA as research on how the organisation of speech creates social activity. The micro analysis in CA is made possible by utilising recordings of actual interaction situations. In CA, there is a strong research tradition of interviews and even interviews particularly in medical settings (see e.g. Ehrling, 2006; Rapley and Antaki, 1998; Simonen, 2017).

Regarding our interviews, we observed how the turns progressed gradually, based on the prior turn. We noticed how the patients’ accounts of their symptoms related closely to the types of question asked. In particular, we were interested in the ways patients’ responses met the expectations created in the clinicians’ questions (Stivers and Hayashi, 2010). During the analysis process 22 extracts were identified, in which the participants oriented differently to the generalisability of personality traits. In these segments, differences between patients’ and clinicians’ reasoning become apparent.

Applying CA to the psychiatric field enables the investigation of psychiatric practices from a sociological point of view. It is like watching the everyday communication in a psychiatric institution from an outsider’s perspective. As we have access to data from actual interactions, we are aiming to understand the participants and the constraints in which they are operating. Whilst we are committed to the conventions in CA, showing the actual talk in detail, we are not committed to either promote or disregard the psychiatry itself. CA is descriptive in its nature, which on the one hand limits the critical power but on the other hand leaves the reader the possibility of forming an own view based on the actual data.

## Results

Below we show extracts that illustrate the different levels of abstraction between questions and answers in our data. However, to give a clear illustration of the interview process, we start with a case in which the patient’s way of answering aligns well with the abstraction level of the question. Then we move on to the more complicated cases. In Extract 1, the patient has filled in a self-assessment questionnaire before the meeting and has responded ‘yes’ to the question: ‘When a close relationship ends, do you feel you immediately have to find someone else to take care of you?’.

Extract 1

01 nu: .hh no sitte ku läheine ihmissuhde päättyy

**.hh well when a close relationship ends**

02  tuntuu et pitää välittömästi löytää

**you feel you immediately have to find**

03  joku muu huolehtimaan t↓ilalle,

**someone else to take care of you as a replacement**,

04 pt:  no mull_o joo sellane et mä_en just siit

**well yeah I have that it’s exactly that**

05  yksin jäämisest tykkää [ni mä oon,]

**I don’t like being left alone [so I have,]**

06 nu:      [◦joo,◦ ]

**[◦yeah,◦ ]**

07 pt:  etin niinku (0.2) korvaa[jan,]

**I’ll find like (0.2) a substitute,**

08 nu:       [elik]kä onks se

**so is it then**

09  niinku tavallaan just se että kunhan

**like in a way that as long as**

10  sun ei tarvi olla yks[in,]

**you don’t have to be alone,**

11 pt:        [kyl]lä.=

**yes.=**

12 nu:  =vai etiksä niiku hoivaajaa .hh tukea

**=or do you look for a caretaker .hh**

13  antavan suhteen [elikkä just,]

**a relationship that offers support [like,]**

14 pt:     [no varmaan ] just se

**[well probably] it’s exactly**

15  et mun ei tarvi olla [yksin,]

**that I don’t have to be [alone,]**

16 nu:     [yksin ] et se

**[alone] so that’s**

17  on se motivaatio joo

**the motivation yeah**

First, the nurse repeats the answer the patient has given in the questionnaire (lines 1–3). Thereby she creates the expectation for the patient to give further information about the matter. The patient explains that he does not like being left alone so he finds a substitute after a relationship ends (lines 4–7). His turn confirms the original answer in the questionnaire and offers some elaboration showing that he understood the idea of the question. His answer also shows that he considers this tendency to be characteristic of his personality in his use of the phrase, ‘I have that’. The participants share the same level of abstraction in their talk: ‘you immediately have to find someone else to take care of you as a replacement’ in the question leads to the description ‘I’ll find like a substitute’ expressed in the answer.

The nurse clarifies the answer in the light of two possible interpretations: that the patient needs the substitute to avoid the feeling of loneliness (lines 8–10) or that he needs to be taken care of (lines 12–13). The patient confirms the first option with ‘yes’ (line 11) and repeats the confirmation after the nurse has presented both options (lines 14–15). The nurse reiterates the central word of the patient’s answer, ‘alone’, showing that they have a mutual understanding of the patient’s behaviour (lines 16–17).

Basing her judgement on the whole SCID-II interview, the nurse concludes in the patient’s medical record that he probably has a borderline personality disorder, in addition to some depressive and paranoid traits.

### Ambiguity and differing perspectives

The question-answer sequences in SCID-II interview do not always flow as smoothly as in the first extract. Next, we show an example that illustrates the complexity of the evaluation process. The participants go through a borderline PD module. In this case, there is no self-assessment questionnaire. The nurse has just asked the question, ‘Is it typical of you to do things on a whim, impulsively?’ The patient has agreed to this, saying that she easily gets excited about new things, but the enthusiasm may quickly disappear. At this point, SCID-II guides the interviewer to investigate the problems caused by the impulsivity. This is where the extract starts.

At the beginning of the interview the nurse has asked the patient to evaluate herself from the perspective of people who know her (this is among the open-ended questions). As she answers the question about the harmfulness of her impulsivity, she evokes this type of approach at her own initiation.

Extract 2

01 nu: .hh mites sitten (.) näyttäytyyks se

**.hh how about then (.) is this ab-**

02 sillä tavalla niiku itselle vahingollisella

**abruptness or or or impulsivity shown in**

03 tai haitallisella tavalla ◦tämmönen◦ (0.4)

**a way that would be harmful or damaging**

04 äk- äkkinäisyys tai tai tai impulsiivisuus tai,

**to yourself or,**

05 pt: kyl se kai myös ja siis niinkun rupesin

**yes perhaps that way too and I mean like I started**

06 miettii tossa: >ku täs_on nyt ollut näit<

**to think about >as there have been several**

07 kysymyksii useempii ja sit oli se et mitä

**of these< questions and there was this that**

08 muut ihmiset [sanois?]

**what other people would [say? ]**

09 nu:        **[mm-m?]**

10 pt: .hh sanois niin tota noin niin #e# tommoset

**.hh would say well so #e# those kind of**

11 hitaamman progression (0.5) tyypit mun ystäväpiiriss↑ä

**people with slower progression (0.5) in my circle of friends**

12 .hh on kokenu tän mun (0.2) äkki- hh jyrkkyydenkin

**.hh have experienced that my (0.2) preci- hh pitousness**

13 toisinaan olevan niinkun .hh öö epäluottamustekijä

**makes me sometimes like .hh less reliable**

14 tai et meil on voinu olla [konf]likteja,

**or that we might have had [conf]licts,**

15 nu:         **[mm, ]**

16 pt: siitä että: et he kokee et tavallaan et

**because they experience in a way that**

17 mä muutun liiankin nopeasti että mihin

**I change too fast so what**

18 he voivat (.) sitten niinkun,

**can they (.) then like,**

19 nu: **mm,**

20 pt: .hh mun olemuksessa nojata=mut et .hh et se

**.hh rely on in my essence=but that .hh that**

21 on ehkä sit niiku tavallaan (0.2) mä näkisin

**it is maybe then like in a way (0.2) I’d see**

22 noi jopa semmosen (0.8) ehkä sit et siel heil

**those even a kind of (0.8) that maybe they have**

23 on joku oma oma (0.2) tämmöne epävarmuus,

**there some of their own (0.2) this kind of insecurity,**

24 nu: **mm,**

25 pt: pohjalla mut et ku mä aina korostan (.)

**deep down but that I always emphasise (.)**

26 sitä että et se että mä oon (0.2) monipuolinen

**that the fact that I’m (0.2) versatile**

27 eläväinen nälkänen ja menevä niin s’ei tee

**lively hungry and energetic that doesn’t**

28 must epärehellist tai epäluotettavaa

**make me dishonest or unreliable**

The nurse asks the patient to evaluate the harmfulness of her impulsivity (lines 1–4). Harmfulness is significant in that it defines whether one could describe the trait as ‘disordered’. The patient partially admits it, saying ‘yes perhaps that way too’. This illustrates the ambiguity of the answer: ‘yes perhaps’ as in making the answer uncertain to begin with and ‘that way too’ as if saying both yes and no. In this way the patient anticipates her departure from the expectations of the question (cf. Niemi, 2014).

The patient recalls how some of her friends ‘with slower progression’ consider her precipitousness a sign of unreliability (lines 10–13). This way she finds circumstances in which she can agree with the question, without truly agreeing. She makes an interesting turn, claiming that perhaps these friends base their views on their own insecurities (lines 20–25). She describes herself in a very positive manner as ‘versatile lively hungry and energetic’, thus showing her disagreement with her friends (lines 25–28), thereby redefining the negative-sounding terms abruptness and impulsivity in the question. Therefore, in the patient’s version, the real problems lie in the insecurity of her friends, whereas she perceives her personality traits as positive and empowering.

As the exchange unfolds further, the patient continues in a similar manner, emphasising the role of her friends and showing the positive quality of her character.

29 pt: vaik mä niinkun .hh mut et et mä oon saanu siit

**even I kind of .hh but that that I’ve received some**

30 joo palautetta mun mun tämmösilt niinkun mh .mt

**feedback about it yes from my these kind of mh .mt**

31 introverteilt hitaamman [progres]sion,

**introvert slow [progres]sion**

32 nu:         **[mmh? ]**

33 pt: tyypeiltä et et niiku et sä (0.5) muutut

**guys that that like you (0.5) change**

34 tosi nopeesti et kyl mä (0.4) mä taas

**very quickly that I (0.4) I on the other hand**

35 oon kokenu sen silleen et mul on nopee

**have experienced it like that I have fast**

36 kasvu(peli),

**growth (game),**

37 nu: **mm-[m,]**

38 pt:   [et]tä: että tota .h mä ↑oon (0.5) toisaalta

**that that I mean .h I am (0.5) on the other hand**

39 valmis: heittämään .hh (.) hienostuneetkin omat

**ready to throw .hh (.) even my sophisticated**

40 uskontojärjestelmäni helvettiin paremman

**belief systems to hell in the face of better**

41 tiedon edessä et [et, ]

**knowledge so that [that,]**

42 nu:      **[◦mm,◦]**

43 pt: et niiku semmonen (5.0) ◦nii◦ mitäs mä taas

**that that like a kind of (5.0) ◦yeah◦ what I now**

44 heh [£tyhjän päälle ajatukseni (kaa)?£]

**heh [£I ended with an empty mind?£ ]**

45 nu:    [joo-o (.) joo-o            ] joo käytännössä

**[yeah (.) yeah            ] yeah in practice**

46 kerroit siitä että .hh >et et< ystäviltä tulee

**you said that .hh friends give you feedback**

47 palautetta mahdollisesti et he ei pysy siinä sun, .hh

**possibly that they do not keep up, .hh**

48 pt: .ts [(–) ja esimerkiks siis,        ]

**.ts [(–) and for example,             ]**

49 nu:      [tah- tahdissa tahdissa mukana (-)]

**[with your pa- pace pace (-)      ]**

At the end of her response the patient expands the theme by bringing up a new perspective (lines 38–41). She mentions another positive side of ‘impulsivity’: being able to adjust her belief systems in the face of better knowledge. Thus, she resists the agenda of the question, which puts the trait in a negative light.

With regard to our claim of ambivalence in the answer, we observe that the patient says ‘yes’ when she talks about the traits causing problems with a certain group of friends (but there is no real reason for this), and ‘no’ in explaining that these traits are not harmful but actually positive qualities in her character.

Finally, the nurse recapitulates her understanding of the answer (lines 45–49). It is interesting that, even though the patient has said that the feedback came from a specific group of friends, the nurse dismisses this information and talks generally about the patient’s friends. The positive aspects of impulsivity the patient refers to are not relevant in the SCID-II framework, and they are not included in the nurse’s summary.

In conclusion, we notice that the level of abstraction varies during the interaction. The nurse starts with a question aimed at evaluating the nature of impulsivity on a general level, to which the patient only offers a context-specific answer. In spite of the patient’s reservations regarding the generalisability of the trait, the nurse concludes that her friends experience her behaviour as problematic. This formulation shows how the nurse brings the interpretation back to a more general level, regarding the patient’s social relations overall.

### The effect of mood and company

In the following case, as in Extract 2, the patient offers a context-specific answer to a general question. In the previous extract, the nurse acknowledged the patient’s answer with minimal response tokens until the final formulation. In this one the nurse poses a second general-level question in response to the patient’s initial contextual answer. The patient maintains his position even after the second question. The participants are going through the obsessive-compulsive module. The patient has responded in the affirmative to the question, ‘Have other people told you that you are stubborn and rigid?’.

Extract 3

01 nu:  mut sit sä oot laittanu et sulle o

**but then you have indicated**

02  et sä oot itsepäine ja jäykkä,

**that you are stubborn and rigid,**

03 pt:  kyllä mä oon sitäki (.) varsinki

**yes I am that too (.) especially**

04  sitte suuttuessani ni (0.2) mä oon

**when I get angry then (0.2) I’m**

05  hyvin itsepäinen,

**very stubborn,**

06 nu:  joo-o mites noin muu:ten ook sä

**yeah how about overall are you**

07 sellanen jäykkä ja [.hh sellanen,]

**a kind of rigid and [.hh kind of,]**

08 pt:          **[mmm,       ]**

09 nu:  jäärä niiku tolleen [noin,]

**headstrong person you know like,**

10 pt:      [riip ]puu

      **[it dep]ends**

11  tilanteesta ja [siitä et,]

**on the situation and on,**

12 nu:    **[mm-m, ]**

13 pt:  mikä hhh mikä on kyseessä et

**the hhh question so that**

14  ammatillisesti mä en oo kauheen .hh #öö#

**in my work I am not very .hh #eh#**

15  jäykkä hh (0.4) asiakkaitani koh[taan, ]

**rigid hh (0.4) with my clie[nts, ]**

16 nu:         [joo-o,]

**[yeah, ]**

17 pt:  asiakkaitamme [.hh mutta,]

**with our clients [.hh but,]**

18 nu:     [nii, ]

     **[yeah, ]**

19 pt:  sitte jotaki kollegaa kohtaan joka

**then I might be very stubborn and**

20  hyökkää niiku vahvasti et hän on

**rigid towards some colleague who**

21  tätä mieltä et nyt tämä pitäis

**attacks in a powerful way saying**

22  hoitaa n↓äin niin ei .hh mä oon sit

**that one should do this thing**

23  hyvin itsepäinen ja jäykkä,

**in certain specific way,**

**24 nu:  .hh**

25 pt:  mut se nyt taas on enemmän mielipide, [.hhh]

**but it is more like an opinion, [.hhh]**

26 nu:        [no] ↑onks

**[well] is there**

27 sellasta et muut olis jotenki sanonu et

**this kind of thing that others would have somehow**

28 sä oot nii:n jäykkä ja itsepäinen että,

**said that you are so: rigid and stubborn that,**

29 pt:  o (.) lapset,

**yeah (.) the kids,**

30 nu: [lapset on sanonu,]

**[the kids have said,]**

31 pt: [hehehe        ] [omat lapset,]

**[hehehe        ] [own kids, ]**

32 nu:         [onks muut ] aikuiset,

        **[have other ] adults,**

The nurse starts by simply stating what the patient has indicated in the questionnaire. The patient has agreed to the assertion that he is stubborn and rigid, but as he starts to elaborate on the answer, the issue seems more complicated. He recognises that he is very stubborn, especially when he gets angry (lines 3–5) – thus, the answer is tied to a specific condition.

The nurse tries again to have a more general description about stubbornness (lines 6–9). However, the patient still emphasises the situational variability (lines 10–13). Here there are signs of tension between the projects of the participants. The patient slightly resists the idea of being able to give the straightforward answer the nurse is looking for. He explains how stubbornness depends on the current social context. We interpret the turn ‘but it’s more like an opinion’ (line 24) as expressing that the patient and his colleagues just have different opinions about how to do things. The patient does not seem willing to call himself rigid in this context; he rather emphasises the difference in views between him and his colleagues. At the end, it does not seem clear that the answer to the original question is positive.

With her persistent questions, the nurse tries to clarify the answer (lines 26–32). She changes tactic and returns to the original question, asking how others would see the patient. The patient admits that his kids have called him rigid (line 29) but this still does not seem to suffice. The nurse would prefer to hear an adult perspective. We notice the difficulty in arriving at a conclusion. Whose perspective is valid enough, and when is the trait pervasive enough?

The nurse concludes in the patient record that there is no indication of a PD. The patient receives an ADHD diagnosis instead.

### Limited generalisability

The new perspective in the next extract is that the patient refers to temporal change. We observe another general-level question posed by the nurse, although a little more specific than the previous ones. Again, the patient describes her behaviour as situationally variable. The participants are the same as in the second extract. The SCID-II interview extended over two meetings in this case, due to the patient’s talkativeness. This extract is from the second interview where the participants deal with obsessive-compulsive traits, and the patient’s recognises some black-and-white thinking. The nurse proceeds with inquiring whether the patient follows the rules very strictly. The extract starts from this follow-up phase.

Extract 4

01 nu: .thh mites sitten: n↑äyttäytyyks tämmönen

**.thh how about then does this kind of (1.0)**

02 niiku (1.0) .mt ääm mustavalkosuus=>ku sä sanoit<

**black-and-white attitude show up >when you said<**

03 et tunneohjautuvuutena mut näyttäy[tyyk-,]

**that in emotion regulation but does this also**

04 pt:       **[mm, ]**

05 nu: tyyks tää my- myös ehkä sellasena että

**show up perhaps in that .hh some**

06 .hh jotkut tietyt säännöt ohjeet lait hh

**specific rules instructions laws hh**

07 on kauheen tarkkoja (0.2) tarkkoja tarkkoja

**are super strict (0.2) strict strict**

08 sum [mie-,]

**in you [min-,]**

09 pt: **     [.thh ]**

10 nu: mielessä tai,

**in your mind or,**

11 pt: .ts mul ↑oli ehkä ↑aikasemmin semmost

**.ts perhaps I used to have more of that**

12 enemmän semmost ehdottomuutta,

**kind of dogmatism before,**

13 nu: **mm,**

14 pt: .hh mutta: nykysellään (2.5) mä_oon vähän

**.hh but nowadays (2.5) I’m a bit like**

15 silleen et kyseenalaista kaikki, .hh

**that I question everything, .hh**

16 nu: nii justiin,

**right,**

17 pt: et ei ei mä oon valmis (.) mä oon valmis muuttaa

**that no no I’m ready (.) I am ready to change**

18 omii näkemyksiini jos tulee vastaan (.) vastaan

**my own views if I encounter (.) encounter**

19 £v(h)akuuttavampaa tietoa£ [tai tai niiku et,]

**more £convincing information£ [or or like     ]**

20 nu:        [aivan joo,        ]

**[yes right,        ]**

21 pt: .hh (0.2) mut s’ei (4.0) .mt mut sit se ehkä

**.hh (0.2) that it doesn’t (4.0) .mt but maybe**

22 tos taitees toi nyt näk[yis vielä,]

**it still shows in that art**,

23 nu:      **[mm,   ]**

24 pt: et jos joku nyt tulis tota noinni .hh (1.0)

**that if somebody now came like .hh (1.0)**

25 mä_en tiä missä vaihees kukaan tulis mun

**I don’t know at what point would anybody come to**

26 prosessin väliin jos jos mun pitäis nyt vaik

**interfere with my process if if I should now**

27 ↑opettaa jotai ihmist piirtä[mään,]

**for example teach somebody to draw,**

28 nu:      **[mm, ]**

29 pt: nii j↑oo siin ois paljon semmosii

**then yes there would be a lot of that kind of**

30 et £t(h)ämä tehdään aina näin?£

**that £this thing is always done this way?£**

31 nu: joo,

**yeah,**

32 pt: .hh mut mut ne on ihan käytännön (.)

**.hh but they are just practical (.)**

33 käytännön semmosii et mul_ei oo semmost

**practical things that I don’t have that kind of**

34 niiku henkist pakkoo siihen [tai,   ]

**mental urge to do that [or,]**

35 nu:         [>aivan,<]

**[>exactly,<]**

36 pt: näin et joku asia tehdään näin [tai,]

**that you do something like this [or,]**

37 nu:           [joo,]

**[yeah,]**

38 pt: saan slaagin .hh öö (0.2)

**I’m gonna lose my nerves .hh eh (0.2)**

The nurse investigates the patient’s black-and-white attitude by asking about strictness in orienting to rules, instructions and laws (lines 1–10). The patient contrasts her earlier and her current character (lines 11–15). She recognises that she has had some dogmatism in her, but not so much anymore; she is now more willing to change her views.

From line 21 on, the patient proceeds to a more specific case. She comes up with a setting that fulfils the expectations of the question: she recognises some strict habits in making art. However, she makes it clear that this is reasonable behaviour in that specific context, so she does not lose her nerves over nothing: ‘they are just practical practical things that I don’t have that kind of mental urge to do that’. The patient does not conceive of this behaviour as generalisable across situations. She briefly continues her turn beyond the last line shown here. The nurse receives patient’s answer by commenting only ‘mm right’ and then moves to the next question. Therefore, we cannot know what she concluded from the answer.

The patient has several points to make both in agreement and disagreement with the question, even to the degree that the answer remains ambiguous. As in Extracts 2 and 3, this one shows a shift from a general-level question to a situational answer. Instead of admitting being strict, the patient refers to temporal change in her personality and context-specific conduct. Both observations imply that the patient does not treat the strictness as an inherent and unchangeable trait. She also points out that she feels fine about this strictness and finds it reasonable, thus contradicting the problematising approach.

On the basis of the SCID-II interview, the nurse notes in the patient record that a borderline PD is likely. Afterwards, a doctor meets the patient and confirms this diagnosis. The obsessive-compulsive PD screened in this extract is not diagnosed.

### Understanding the behaviour

Next, we show a case in which the question is more specific than in the previous extracts, referring to conduct rather than an inner trait. The patient nevertheless moves towards a more specific case. The participants go through the SCID-II module that concerns antisocial traits. One of the diagnostic criteria for antisocial personality disorder is running away from home and staying away overnight before the age of 15. Even though the question is about specific conduct, it still treats the behaviour as indicative of an inner trait of indifference to other people and common rules.

Extract 5

01 nu:  entä sitte kark↑ailit sä kotona?

**and did you have a habit of running away from home?**

02  (2.0)

03 pt:  tota: .mt jos mä näin et se tilanne on

**well .mt if I saw that the situation is**

04  ahdistava, [hh ]

**pressing,  [hh ]**

05 nu:        **[mh,]**

06 pt:  niin kyllä mä sit mull_oli mönkijä ni

**then yes I did I had a quadbike so**

07  mä (.) kävin aika paljon tossa (.) <nuor->

**I (.) went quite a lot there (.) <you->**

08  siinä tota (0.2) turvatalossa,

**to that (0.2) youth shelter,**

**09 nu:  mm-m,**

10 pt:  lähin sinne koska mä en sietäny sitä

**I went there because I couldn’t stand that**

11  tilannetta,

**situation,**

12 nu:  joo,

**yeah,**

13 pt:  ↑enkä välttämättä mä vaan lähin jos

**and I didn’t necessarily I just took off if**

14  mä näin (.) et isä on nyt tappelee mun

**I saw (.) that dad is now fighting with my**

15  veljen kanssa,

**brother,**

16 nu:  joo,

**yes,**

17 pt:  sitä sitä ei kiinnosta ollenkaan nyt

**he he is not interested now at all**

18  jos mä=lähen=ni sitte mä olin s↑illain

**if I=go=so then I was like**

19  et joo (1.0) mä vaan sanoin sinne

**yeah (1.0) I just said to the**

20  turvataloon et voit sä s↑oittaa sille

**shelter that you can call him**

21  mutta ei sitä tuskin kiinno- tuskin

**but he is hardly interes- hardly**

22  kiinnostaa että,

**interested that,**

23 nu:  ja näihin oli [tie]tyt,

**and there were certain,**

24 pt:      **[mm,]**

25 nu:  syyt että [ul ]koiset,

**reasons so [ou]ter,**

26 pt:      **[mm,]**

27 nu:  syyt jotka tuotti sit sen [et sä,]

**reasons that caused you to,**

28 pt:        **[mm, ]**

29 nu:  lähdit tur[vaan,]

**seek shelter,**

30 pt:      [joo, ]

**[yeah,]**

**(Two extra questions omitted)**

39 nu:  et (.) okei nää oli tämmösiä,

**you didn’t (.) okey these were these kind of,**

40 pt:  joo,

**yeah,**

41 nu:  asosiaalisuu[teen liittyviä piirteitä,]

**traits associated with asociality,**

42 pt:      [joo en (kyl -)        ]

**[yeah I (didn’t -)        ]**

43 nu:  käytännössä nyt sit tän meiän

**in practice now based on this**

44  haastattelun perusteella niin niin

**our interview so so**

45  sellasta niinkun vahvaa (0.8) selkeästi

**there is not that kind of strong**

46  mihinkään [per ]soonallisuushäiriöön,

**marker of any personality disorder,**

47 pt:     [joo,]

**[yeah,]**

48 nu:  viittavaa ei [ei,]

**coming up,**

49 pt:       **[mm,]**

50 nu:  näy ja sillä mä käyn nää vielä läpi,

**and I will still go through these,**

The formulation of the question refers to a regular habit. After a 2-second pause, the patient offers a positive answer. It is noteworthy, however, that she starts with explaining her behaviour (lines 3–4) and only then does she indicate agreement, ‘yes I did’ (line 6). In this way the patient puts the emphasis on the explanation. She continues by describing the circumstances more precisely. She ties her description to social relations: first explaining that the fights were the reason for running away, and then elaborating on the interaction at the shelter.

During lines 23–31, the nurse formulates the relevant point of the answer: that there were certain outer reasons that caused the patient to seek shelter. After that, she continues with a few more diagnostic questions (data not shown), and then states her overall view that the patient does not seem to have strong markers of any personality disorder (lines 43–46). It appears from her short affirmations that the patient agrees with the conclusion.

In this extract, the question and the answer operate almost at the same level of abstraction. However, as noted above, the question projects a more generalised view of personality. In explaining that she was running away from home to protect herself, the patient is still on more specific level. Although she admits running away, the nurse treats her response as being somewhat negative. She seems to accept and understand patient’s explanation, and does not attempt to generalise the patient’s behaviour as an inherent trait.

The interview leads to the conclusion that the patient does not have a PD. The diagnosis of undefined anxiety remains in the patient record.

### The patients’ reasoning versus SCID-II

The extracts discussed in this section are quite representative of the 22 cases regarding different approaches of a clinician and a patient to making generalisations about behaviour. Furthermore, having broken down our observations we make the following points.

(1)  Patients frequently make sense of their own behaviour differently than SCID-II predicts – not as a reflection of their inner stable personality traits, but as outcomes of many situational factors.
(2)  Patients hardly ever straightforwardly oppose the format of the questions. However, their responses sometimes go against the underlying assumptions.
(3)  When patients exclude personality in explaining their behaviour, they probably refer to the social context.
(4)  Patients sometimes refer to acute inner states as important factors affecting their behaviour. The difference from SCID-II expectations is that inner states vary constantly, and thus patients do not see the behaviour as being generalisable to other situations involving a different mindset.
(5)  Patients might also think that behaviour touches only one small area of life and cannot be generalised based on that. In many cases they are able to come up with a context in which they behave as asked, but they would not see this as describing their overall personality.
(6)  Patients do not necessarily think of their personality as remaining stable across adulthood, but they might notice changes over time and as a result of life experiences.
(7)  The questions vary in their level of abstraction, but patients frequently take a step towards a more situational level.

## Discussion

We have looked closely at PD interviews, with a specific focus on moments of discrepancy between clinician and patient regarding the generalisability of behaviour. Given that the psychiatric diagnostic system is rather vague and open to interpretation (Vanheule, 2017), it is important to highlight the processes that lead to a diagnosis.

We have illustrated how the ‘voice of the life world’ and the ‘voice of medicine’ (Mishler, 1984) meet in PD assessment. We have shown some problematic patterns in which the assumption of de-contextualised symptoms become challenged. Our observations show that patients often emphasise contextual over generalising factors. This explanatory model is sometimes in conflict with SCID-II, which seeks for inherent and long-lasting traits that cause certain behaviour. We have observed how clinicians operate within medically oriented psychiatry, and thus need to isolate the patient’s conduct from the contextual variables before making their evaluations.

Our study still leaves open how much patients’ unwillingness to generalise the traits to describe themselves is linked to the negative personal qualities that the questions are about, such as being a rigid and stubborn person. It would be interesting to discover how discussing about positive personality traits might change the results; would patients be more inclined to generalise positive-sounding qualities to describe their personality overall?

Given the limited amount of data, the generalisability of the findings remains uncertain. It is possible that some of them relate only to individual participants and do not represent psychiatric evaluation in general. However, we collected our data in an ordinary outpatient clinic following rather unified assessment procedures, and therefore it is still quite probable that the results represent normal practices.

Our data comes from a clinic where the psychiatric nurses are responsible for the diagnostic screening for PDs. It would be worth investigating this shift of work from psychiatrists to nurses. As we noticed in our research, the protocol still leaves a lot of space for the influence and the interpretations of the interviewer. It is also an interesting point of view that psychiatrists are nevertheless responsible for the diagnosis, despite their lack of involvement in the assessment process.

Given the aforementioned unclarity regarding PD diagnoses, more understanding about their essence is required. For instance, PDs correlate with race and gender (Manning, 2000) – how are we to understand these aspects? There is also a need to investigate the clinical practises on how conditions are being transformed into formal diagnoses and treatment plans (Duff et al., 2020). For the sake of clarity, we separate two levels of uncertainty regarding PD diagnoses. On the root level we have a question of the construct itself: the scientific basis for PD categories, the overlap between them and other psychiatric symptoms, the fundamental question of why certain personality traits are being held as disorders in our society and where the line is drawn between normal and pathological personality. On the second level there is an issue with the diagnostic process: its objectivity, the interaction where the picture of the condition is being constructed, the reasoning of the participants, and the institutional underpinnings of the interviews. This paper is concerned with the latter, but of course these levels are largely interconnected.

Our results support Georgaca’s (2013) observations on psychiatric formulations. She suggests that clinicians are selective in the diagnostic process to ensure that patients’ complaints fit into the psychiatric model. We observed, for instance, that clinicians left out positive self-evaluations by patients from the formulation because it has no place in diagnostic assessment.

However, SCID-II treats interview answers as an accurate description of the patient apart from the assessment situation. Constructionist tradition has criticised this kind of view on interviews. It emphasises that data should rather be regarded as a topic itself, reflecting a reality constructed in collaboration (Rapley, 2001). Regarding psychiatric interviews, Ribeiro and de Souza Pinto (2006) state that stories should be viewed as situational discourses, being contingent on the context. Interestingly, clinicians rarely talk about the ontological and epistemic uncertainty of the psychiatric diagnoses with patients, thus treating the diagnoses as real objects (Lane, 2020).

According to McGann (2011), symptom checklists actually make it more difficult to evaluate a patient’s meaningful reactions because they detach symptoms from their contexts. Answering PD questions in the affirmative would imply inappropriate reactions. However, the interview protocol does not create an understanding of the dynamics of behaviour. In a psychiatric framework, distress can be constructed as an independent entity and thus be isolated from the sufferer (LaFrance, 2007). We question the meaningfulness of this kind of isolation.

In our research, we have no causal data of the effects of the interview format on any outcomes. We can only observe how a patient’s formulation becomes problematic in the SCID-II interview framework, and part of the answer needs to be either reformulated or ignored. Based on our observations, it is easy to align with the view of Savander et al. (2019) that by facilitating patients’ opportunities to reveal their subjective experiences, there is a chance to build shared understanding of patients’ unique situations and improve more individualised care planning. Evidence from medical settings shows that orienting together more to the lifeworld approach results in better outcomes and more humane treatments (Barry et al., 2001).

As an alternative to the current diagnostic approach, there would be a chance to use case formulations that regard patients’ descriptions about the impact of their social environment and situational variability as relevant information (e.g. Vanheule, 2017). The idea is to withdraw from symptom checklists and create descriptions that more accurately capture psychological dynamics in individuals (Savander et al., 2019).

We wish to highlight in particular the importance of showing real interactional phenomena during psychiatric assessment, which will facilitate a more insightful evaluation of them. Karter (2019) emphasises the importance of a critical understanding of psychiatric diagnosis among mental-health professionals. He argues that humility in the face of limits of knowledge would strengthen the ethicality of the work. Critical awareness would include understanding how different institutions shape the constructs of disorders and manuals, and of the possible consequences of a certain diagnosis for the patient. We would like to think that our research increases the likelihood for developing such awareness.

As Tyrer et al. (2007) state: ‘Just as mental state can be dependent on environmental influences, so can personality status’. They explain that personality works differently in different ages and as a response to varying demands. Despite the moderate stability of traits, no absolute tendency exists that would predict a clear prognosis. Luyten et al. (2020) claim that the perceived rigidity and stability of ‘personality’ in PDs is more like an adaptation strategy. According to them, the approach of attributing the rigidity simply to the individual is misleading: one should observe the type of relationship the patients have with others and their environment. PD actually reflects a disorder of social communication, and that its stability lasts only as long as the underlying mechanisms are active (Luyten et al., 2020).

The system for diagnosing PDs will change dramatically in the future, as the trait continuum will replace the categorical system of ICD-11. This transformation seems welcome as it reflects better the current understanding of personality, and it could also enable more individualised personality formulations. How the adaptation of the new system will happen in psychiatry remains to be seen. It will be essential to research the application of this new system on a practical level.
